# Geometric Optimization of GMR Biosensors with Trapezoidal Magnetic Flux Concentrators for Detecting *Bacillus anthracis* in Complex Matrices

**DOI:** 10.3390/s26082424

**Published:** 2026-04-15

**Authors:** Changhui Zhao, Jiao Li, Hao Sun, Chunming Ren, Shenghao Li, Chong Lei, Zhen Yang, Xuecheng Sun

**Affiliations:** 1 Microelectronics Research & Development Center, School of Mechatronics Engineering and Automation, Shanghai University, Shanghai 200444, China; zhaochanghui2023@shu.edu.cn (C.Z.); lijiaoshu@shu.edu.cn (J.L.); sun_hao@shu.edu.cn (H.S.); rcm@shu.edu.cn (C.R.); 19155520102@shu.edu.cn (S.L.); 2 National Key Laboratory of Advanced Micro and Nano Manufacture Technology, Department of Micro-Nano Electronics, School of Electronic Information and Electrical Engineering, Shanghai Jiao Tong University, Dongchuan Road 800, Shanghai 200240, China; leiqhd@sjtu.edu.cn; 3 College of Electronic and Information Engineering, Guangxi Normal University, Guilin 541004, China; 4 Shanghai Key Laboratory of Automotive Intelligent Network Interaction Chip and System, The School of Microelectronics, Shanghai University, Shanghai 200444, China

**Keywords:** GMR biosensor, *Bacillus anthracis*, magnetic flux concentrator (MFC), finite element magnetic simulation, complex matrices

## Abstract

Background noise and intensive sample preparation frequently compromise the field screening of *Bacillus anthracis*. Addressing these analytical bottlenecks, we constructed a giant magnetoresistive (GMR) biosensor incorporating geometrically tailored trapezoidal magnetic flux concentrators (MFCs). 3D finite element magnetic simulations directed the MFC topology to mitigate edge saturation, reconciling central magnetic gain with spatial uniformity. The resulting platform demonstrated a 100-fold sensitivity improvement over recent electrochemical methods, achieving a limit of detection (LOD) of 10 CFU/mL in standard buffers, with the entire testing process completed within 40 min. Direct target quantification remained viable in heterogeneous matrices—muddy water, whole milk, and apple cider—circumventing tedious pretreatment. This geometric and magnetic optimization yields a pragmatic sensing architecture tailored for on-site biodefense monitoring.

## 1. Introduction

Classified as a Tier 1 biological agent, *Bacillus anthracis* presents persistent public health challenges, primarily driven by the extreme environmental resilience of its spores and the acute mortality rate associated with inhalation anthrax [1,2,3,4]. Advancing current biodefense frameworks now largely depends on achieving rapid, high-sensitivity screening within complex matrices—such as contaminated soils or agricultural food chains—where background interference often complicates conventional detection methods [5,6].

Current detection methodologies typically encounter distinct operational bottlenecks in field settings. Although molecular assays such as Polymerase Chain Reaction (PCR) and CRISPR systems achieve exceptional sensitivity [7,8,9], their dependence on benchtop instrumentation and intricate nucleic acid extraction protocols restricts their translation into point-of-care testing (POCT) [10,11,12]. Lateral flow assays (LFAs) address the demand for portability, yet they generally exhibit suboptimal detection thresholds [13], elevating the risk of false negatives during trace-level screening. Matrix interference presents another persistent challenge for conventional optical and electrochemical platforms. Extracting reliable target signals directly from crude mixtures such as mud or milk [10,14,15,16,17,18] remains notoriously difficult, as the readouts are inherently obscured by physical light scattering and electrochemical Debye shielding.

By exploiting the inherent “magnetic transparency” of biological matrices, giant magnetoresistive (GMR) biosensors bypass the need for extensive sample pretreatment, enabling direct target quantification even in highly opaque environments [19,20,21,22]. Building upon our group’s previous work utilizing magnetic flux concentrators (MFCs) to amplify localized signals [23], further characterization reveals that standard rectangular MFC geometries remain susceptible to “edge saturation” under external excitation [23,24,25,26]. This saturation constrains the maximum achievable magnetic gain while simultaneously inducing spatial field heterogeneity across the sensing interface. Overcoming this physical bottleneck necessitates a deliberate topological redesign of the MFC structure to better reconcile peak magnetic gain with spatial field uniformity [27,28].

Addressing this geometric limitation, we developed a GMR biosensor integrating optimized trapezoidal MFCs. By utilizing 3D finite element magnetic simulations in COMSOL, the modified topology was designed to mitigate edge saturation, thereby establishing a more favorable equilibrium between central magnetic gain and field uniformity. The analytical performance was subsequently evaluated through a double-antibody sandwich immunoassay targeting inactivated *B. anthracis*. The proposed platform demonstrated a limit of detection (LOD) of 10 CFU/mL in a standard PBS buffer. Extending the assay to simulate field sampling, the sensor directly quantified target bacteria concentrations as low as 50 CFU/mL in high-interference matrices—including muddy water, whole milk, and apple cider—bypassing conventional pretreatment workflows. The consistent signal recovery across these complex backgrounds indicates the viability of the trapezoidal GMR configuration for decentralized biodefense screening.

## 2. Materials and Methods

### 2.1. Bacterial Strains, Reagents, and Materials

Comprehensive details of the biological and chemical reagents are provided in Table 1. The double-antibody sandwich immunoassay was constructed utilizing inactivated *Bacillus anthracis* suspensions (ranging from $10^{1}$ to $10^{4}$ CFU/mL) and matching specific antibody pairs, while introducing distinct non-target bacterial suspensions to profile the assay’s cross-reactivity. Operators executed all microbiological steps strictly within a certified Biosafety Level 2 (BSL-2) laboratory.

For signal amplification, streptavidin-coated superparamagnetic nanobeads (Dynabeads™ MyOne™ C1, 1.0 μm diameter, Thermo Fisher Scientific, Waltham, MA, USA) functioned as magnetic labels. Surface functionalization relied on analytical-grade reagents, including 11-MUA, EDC, and NHS.

To assess the sensor’s tolerance to matrix interference, testing environments were modeled using whole milk, acidic apple cider (NFC juice), and environmental mud. The mud suspensions were prepared by homogenizing soil in deionized water (1:1 *w*/*w*) and passing the mixture through a 2 mm sieve. These native matrices were subsequently spiked with the target pathogen, closely emulating genuine field contamination scenarios.

### 2.2. 3D Finite Element Magnetic Simulation

To optimize the geometric parameters of the MFCs, three-dimensional (3D) finite element magnetic simulations were performed using the COMSOL Multiphysics software (Version 6.0, COMSOL Inc., Stockholm, Sweden). The Magnetic Fields (mf) interface within the AC/DC Module was employed to calculate the spatial distribution of the magnetic flux density.

**Figure 1:**
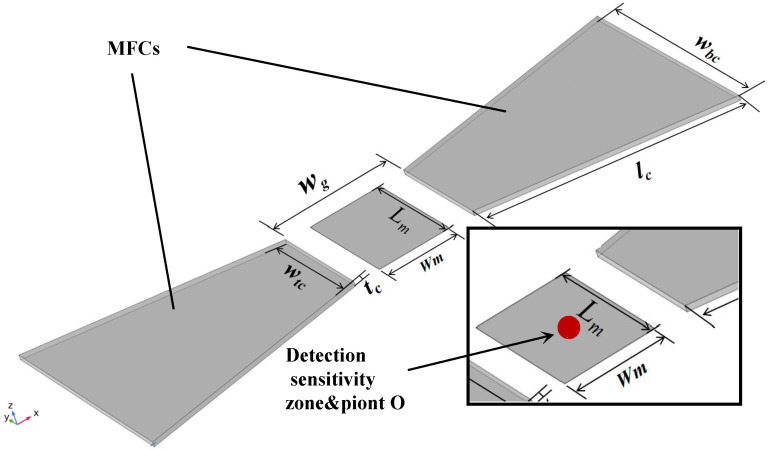
Three-dimensional finite element simulation model of the GMR biosensor integrated with trapezoidal magnetic flux concentrators (MFCs).

The geometric model is shown in Figure 1. To evaluate the flux concentration efficiency in the low-field linear region, a uniform external bias field ($B_{\mathrm{ext}}$) of 10 Oe was applied along the *X*-axis at the air domain boundaries. In the simulation environment, the MFC domain was assigned a constant relative permeability ($\mu_{r}$) of 1000, representing a standard isotropic soft magnetic medium (e.g., Nickel). This idealized baseline effectively isolates the geometric shape anisotropy from complex material-specific nonlinearities, thereby streamlining the structural optimization process [26].

The dimensional parameters of the initial model are summarized in Table 2. During the design progression, the dimensions of the active GMR sensing region ($L_{m}$ and $W_{m}$) were finalized in the ultimate layout stage to enlarge the biochemical capture area and match the target base resistance. Drawing upon the extensive experimental expertise accumulated by our research group, several MFC geometric parameters were predetermined as optimized constants to ensure both device performance and fabrication reliability. Specifically, based on verified fabrication protocols, the sensing gap ($w_{g}$) was fixed at 450 μm to ensure sufficient lithography alignment tolerance and prevent physical short-circuiting over the GMR stripes. The MFC thickness ($t_{c}=2\;\mu m$) was set to an empirical optimum that maintains the mechanical stability of sputtered films, and the bottom width ($w_{\mathrm{bc}}=1000\;\mu m$) was kept constant as a standardized baseline for environmental flux collection.

To systematically evaluate the performance of the MFC geometries under these physical constraints, two key indicators were defined. The first is the central gain *G*. It is defined as the ratio of the magnetic flux density $B_{\mathrm{center}}$ at the gap’s geometric center to the external field $B_{\mathrm{ext}}$:

(1) $$G=\frac{B_{\mathrm{center}}}{B_{\mathrm{ext}}}$$

To prevent spatial field heterogeneity from distorting the sensor’s transfer curve, a magnetic field homogeneity index ($H_{\mathrm{index}}$) was incorporated to quantify this physical constraint [27]. Establishing the air gap center gain ($G_{0}$) as the baseline, this parameter demarcates an “effective uniform region” along the central *Y*-axis (denoted as $L_{\mathrm{uni}}$). Within this specific spatial window, the local gain fluctuation is strictly constrained between $0.99G_{0}$ and $1.01G_{0}$ (corresponding to a deviation of ≤1%). We measure the spatial uniformity using $H_{\mathrm{index}}$, which represents the fraction of the total active sensing area $L_{\mathrm{total}}$ occupied by this effective uniform region $L_{\mathrm{uni}}$:

(2) $$H_{\mathrm{index}}=\frac{L_{\mathrm{uni}}}{L_{\mathrm{total}}}\times 100\%$$

Physically, an elevated $H_{\mathrm{index}}$ signifies that a greater percentage of the designed sensing area ($L_{\mathrm{total}}$) functions within a homogenized bias field. This expanded uniform coverage effectively mitigates nonlinear signal distortion, thereby ensuring robust measurement reproducibility across the sensor.

Having established the fixed fabrication boundaries and the dual performance metrics (*G* and $H_{\mathrm{index}}$), the optimization process strictly focuses on the remaining tunable degrees of freedom: the top width ($w_{\mathrm{tc}}$) and the longitudinal length ($l_{c}$). From a physical perspective, these two parameters govern the shape anisotropy and the reluctance of the magnetic path. Specifically, $l_{c}$ determines the effective volume available to capture the ambient magnetic flux, which dictates the theoretical ceiling of the magnetic gain. Concurrently, $w_{\mathrm{tc}}$ controls the convergence angle of the magnetic flux lines as they are funneled into the sensing gap. While a narrower $w_{\mathrm{tc}}$ sharply concentrates the magnetic lines to amplify the local gain (*G*), it inevitably exacerbates fringing fields at the gap edges, severely deteriorating the field uniformity ($H_{\mathrm{index}}$) across the GMR sensor array. Because these two parameters induce a direct physical trade-off between signal amplification and spatial homogeneity, the subsequent simulations systematically sweep $w_{\mathrm{tc}}$ and $l_{c}$ to map their competing impacts on the magnetic field distribution, ultimately deriving an optimal geometric balance that satisfies both high sensitivity and excellent spatial uniformity.

### 2.3. Sensor Fabrication

**Figure 2:**
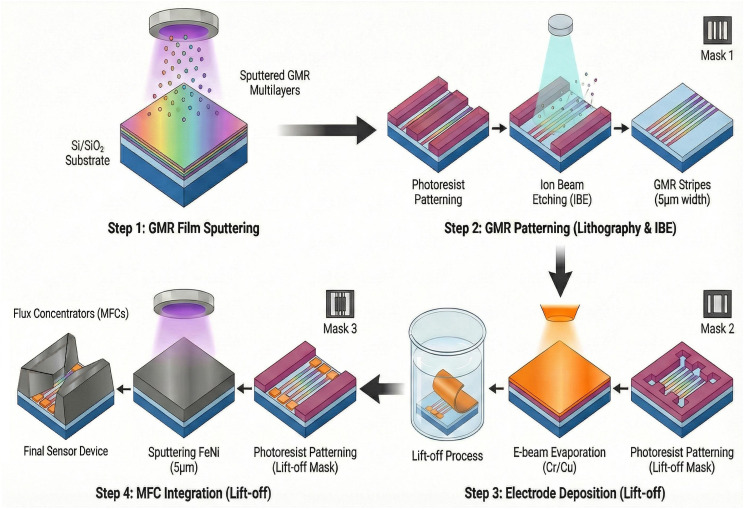
Schematic illustration of the microfabrication process flow for the GMR biosensor.

The microfabrication process flow for the GMR biosensor, built on 6-inch thermally oxidized silicon (Si/SiO_2_) wafers, is illustrated in Figure 2. The procedure comprises four main stages: GMR film sputtering, stripe patterning, electrode deposition, and MFC integration. The core fabrication steps followed our previously optimized protocol [23].

In Step 1, the bottom-pinned spin-valve stack was deposited via high-vacuum magnetron sputtering with the following layer sequence (thicknesses in nanometers): Ta (5)/FeNi (2)/IrMn (8)/CoFe (2)/Ru (0.8)/CoFe (2)/Cu (2.3)/CoFe (1.5)/FeNi (2)/Ta (3). The antiferromagnetic IrMn layer pins the adjacent ferromagnetic reference layer to establish the pinning field direction. The rectangular GMR stripes possess a shape anisotropy that defines the easy magnetization axis along their longitudinal direction. During subsequent testing, the external magnetic field is applied along the transverse hard axis (orthogonal to the pinning direction) to ensure a linear and hysteresis-free transfer curve.

In Step 2, the stack was patterned by ion beam etching (IBE) to form the active sensing area. To enhance the sensor’s sensitivity to weak magnetic field variations, the sensing unit was designed utilizing GMR stripes with a high length-to-width ratio. Specifically, the stripes were patterned with a line width of 5μm and a spacing of 10μm, resulting in an effective active area of 800μm×900μm. Based on this specific layout, the theoretical zero-field base resistance of the sensor is designed to be approximately 9.6kΩ.

In Step 3, the Cr/Cu interconnects (capped with 150 nm of Au for electrical contact) and a 200 nm thick SiO_2_ passivation layer were structured via electron-beam evaporation and a standard lift-off process.

Finally, in Step 4, trapezoidal MFCs were integrated to overcome the edge saturation inherent in conventional rectangular designs. In our simulations (Section 2.2), we used an idealized linear magnetic medium ($\mu_{r}=1000$, representing Ni) to isolate the effects of geometric shape anisotropy from complex material nonlinearities. For the actual device fabrication, we chose Permalloy (FeNi). Because the relative permeabilities of both materials are vastly greater than air ($\mu_{r}\gg 1$), the spatial distribution of the magnetic flux is dictated almost entirely by the geometric demagnetizing factor, not the specific permeability value. Consequently, the geometric optimization rules established in the simulation hold true for the fabricated devices. Moving to Permalloy in practice offers additional physical advantages. Its significantly higher initial permeability ($\mu_{r}>1000$) directly enhances the absolute magnetic gain. Additionally, Permalloy’s near-zero magnetostriction and ultra-low coercivity are practically required to prevent the 2 μm thick sputtered film from peeling due to internal stress, while also ensuring the sensor operates without magnetic hysteresis.

### 2.4. Surface Functionalization and Detection Protocol

**Figure 3:**
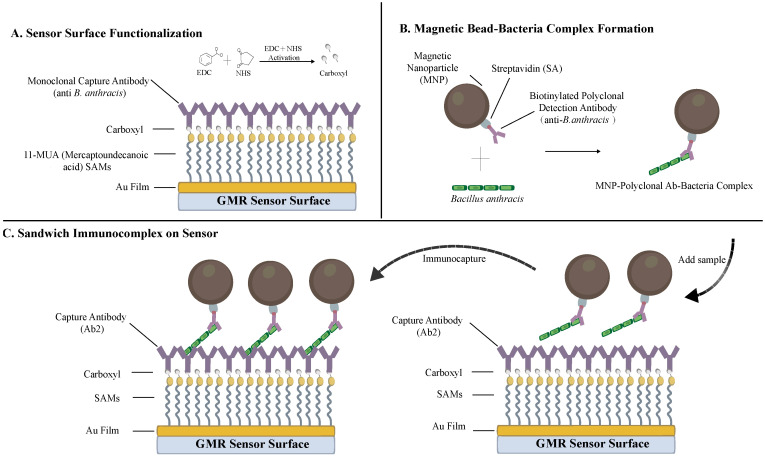
Schematic illustration of the surface functionalization and immunocapture detection principle.

The immunocapture interface was constructed on the top Au film of the GMR sensor utilizing a covalent self-assembled monolayer (SAM) strategy (Figure 3). Pretreatment of the Au surface, the formation of the 11-MUA SAM, and the subsequent EDC/NHS carboxyl activation were executed by adapting our previously established protocols [23,29]. Upon establishing this reactive layer, pathogen-specific monoclonal capture antibodies against *Bacillus anthracis* were covalently immobilized onto the sensing region, and the unreacted sites were subsequently quenched with 1% bovine serum albumin (BSA).

For signal amplification, the platform employed a double-antibody sandwich configuration utilizing streptavidin-coated magnetic nanobeads (MNPs). To maintain biosafety compliance, target *B. anthracis* samples were derived exclusively from inactivated bacterial suspensions. During the assay, a 5 μL droplet of the pre-conjugated “MNP-pAb-Bacteria” complex was dispensed onto the functionalized sensor for on-chip incubation. This setup was incubated in a water bath incubator at 37 °C for 30 min to drive the immunocapture kinetics.

To isolate the specific binding events, residual unreacted components were flushed away with PBS for 5 min. The resulting variations in magnetoresistance, directly driven by the anchored magnetic labels, were then captured via a laboratory-built testing suite. All measurements were performed at least in triplicate. Excluding the off-chip sensor functionalization, the entire on-chip detection workflow—encompassing sample incubation (30 min), washing (5 min), and signal acquisition (<5 min)—was completed within 40 min.

### 2.5. Measurement Setup and Signal Processing

**Figure 4:**
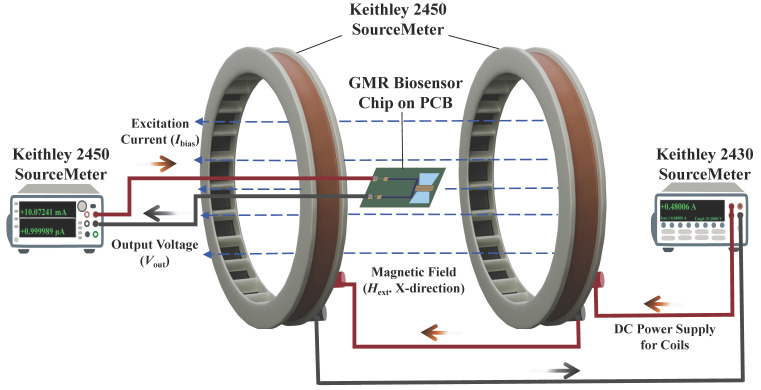
Schematic diagram of the high-sensitivity magnetic measurement platform.

The magneto-electrical characterization of the GMR biosensors was performed on a custom-built platform (Figure 4). Building upon our previously validated hardware configuration [23], the measurement system utilizes homemade Helmholtz coils driven by a Keithley 2430 SourceMeter to generate the required uniform DC magnetic field. This specific coil configuration is critical as it generates a highly uniform magnetic field across the central testing area, thereby eliminating measurement errors caused by spatial magnetic field gradients. For signal acquisition, a Keithley 2450 SourceMeter (Keithley Instruments, Cleveland, OH, USA) supplied a continuous 1 μA excitation current to the sensor while monitoring the dynamic voltage output.

Instrument synchronization and data logging were managed by a custom Python (version 3.9) script via USB interfaces. This automated setup supports high-frequency data sampling and real-time signal visualization. Fundamentally, the magnetoresistance ratio (MR) originates from the spin-dependent scattering of conduction electrons within the multilayer stack, which is physically defined by Equation (3):

(3) $$\mathrm{MR}=\frac{R_{\mathrm{ap}}-R_{p}}{R_{p}}$$

where $R_{\mathrm{ap}}$ and $R_{p}$ represent the electrical resistances of the GMR sensor when the magnetizations of the free and reference layers are in an antiparallel and parallel alignment, respectively. Under a constant excitation current, this intrinsic resistance variation is transduced into a measurable voltage shift according to Ohm’s law. Based on the acquired raw voltage data, the experimental MR was calculated as expressed in Equation (4):

(4) $$\mathrm{MR}\left(\%\right)=\frac{R_{\mathrm{ext}}-R_{\mathrm{init}}}{R_{\mathrm{init}}}\times 100\%=\frac{V_{\mathrm{ext}}-V_{\mathrm{init}}}{V_{\mathrm{init}}}\times 100\%$$

Here, the parameters $V_{\mathrm{ext}}$ and $R_{\mathrm{ext}}$ define the sensor’s voltage and resistance during external magnetic excitation. $V_{\mathrm{init}}$ and $R_{\mathrm{init}}$ represent the initial baseline values in a zero-field state.

Upon successful target capture, the immobilized magnetic nanoparticles (MNPs) project a localized stray field that counteracts the applied bias. This magnetic superimposition effectively attenuates the net field penetrating the GMR elements, thereby inducing a measurable decrease in device resistance. To mathematically correlate this physical shift with the biological binding events, the target-induced signal (ΔMR) is formulated as Equation (5):

(5) $$\Delta \mathrm{MR}={\mathrm{MR}}_{\mathrm{pre}}-{\mathrm{MR}}_{\mathrm{post}}$$

In this signal model, ${\mathrm{MR}}_{\mathrm{pre}}$ establishes the stable initial magnetoresistance ratio of the sensor under a bias field prior to sample introduction, whereas ${\mathrm{MR}}_{\mathrm{post}}$ represents the final ratio recorded by the sensor under an identical bias field following the complete assembly of the immunocomplexes. The resulting ΔMR reflects the quantity of captured magnetic labels, providing a deterministic basis for the quantitative assessment of the target bacterial concentration.

## 3. Results and Discussion

### 3.1. Simulation and Geometric Optimization

**Figure 5:**
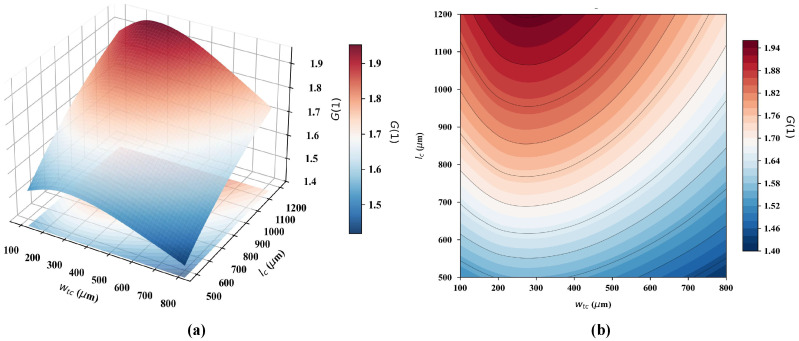
Simulation results of the magnetic field gain for trapezoidal MFCs. (**a**) 3D response surface plot of gain *G* versus $w_{\mathrm{tc}}$ and $l_{c}$. (**b**) Corresponding 2D contour plot.

To optimize the trapezoidal MFC topology, 3D computational modeling was conducted using COMSOL Multiphysics to evaluate flux-concentrating efficiency and to mitigate the severe edge saturation inherent in rectangular geometries [23]. We systematically characterized the synergistic influence of the top width ($w_{\mathrm{tc}}$) and longitudinal length ($l_{c}$) on the central magnetic gain (Figure 5).

As illustrated in Figure 5a,b, extending the longitudinal length $l_{c}$ correlates positively with the magnetic field gain, as a larger MFC footprint captures flux lines over a broader spatial volume before funneling them into the central air gap. Despite this performance advantage, considerations regarding biochip integration and fabrication costs necessitated a practical limit on the device footprint. Thus, $l_{c}$ was fixed at 1100 μm—a dimension that yields a high-gain output near the saturation threshold. In contrast, the top width $w_{\mathrm{tc}}$ exhibited a non-monotonic impact on gain performance; Peak magnetic gain emerged at narrower $w_{\mathrm{tc}}$ dimensions, further widening progressively diluted the essential flux-funneling mechanism.

**Figure 6:**
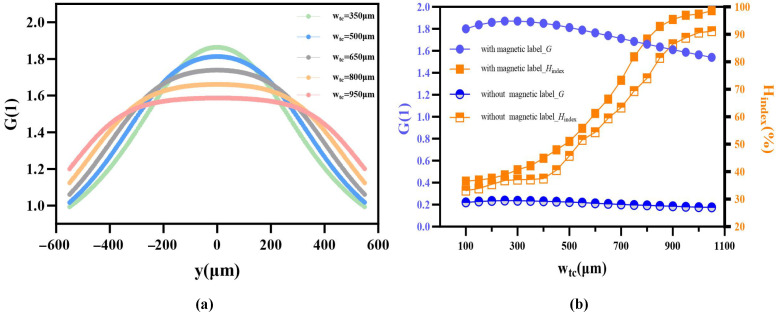
Magnetic field uniformity analysis and geometric optimization. (**a**) Induced magnetic field gain curves along the central *y*-axis of the sensing gap. (**b**) Dual-axis trade-off analysis between central gain *G* and uniformity range ratio.

Spatial uniformity across the sensing gap is as critical as absolute magnetic gain for maintaining the linear response of the GMR elements. Profiles extracted along the central *y*-axis (Figure 6a) reveal that narrow $w_{\mathrm{tc}}$ configurations yield a sharp, “bell-shaped” distribution. While providing high peak gain, such geometries suffer from rapid spatial decay, restricting the effective uniform sensing region. Increasing $w_{\mathrm{tc}}$ to 950 μm causes the distribution to broaden into a desirable “flat-top profile,” indicating a substantial improvement in field homogeneity.

To balance sensitivity against uniformity, a trade-off analysis was performed by evaluating the central gain (*G*) and the homogeneity index ($H_{\mathrm{index}}$) via Equations (1) and (2). As summarized in Figure 6b, expanding $w_{\mathrm{tc}}$ drives a significant upward trend in $H_{\mathrm{index}}$ at the cost of a gradual, manageable decline in central gain. To ensure that magnetic labels immobilized within the active detection zone experience a maximally uniform excitation field, $l_{c}=1100$μm and $w_{\mathrm{tc}}=750$μm were identified as the optimal topological parameters. This configuration achieves a substantial magnetic gain of 1.69 while maintaining an $H_{\mathrm{index}}$ of 81.8%, effectively integrating high signal intensity with a broad linear operating range.

### 3.2. Evaluation of Magnetoresistive Performance

**Figure 7:**
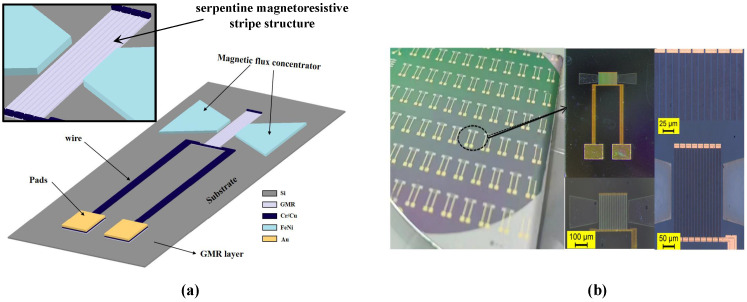
3D schematic illustrations and optical images of the GMR sensor. (**a**) 3D schematic illustration of the sensor structure and layer composition. (**b**) Optical microscope image of the fabricated sensor array.

To validate the microfabrication quality, the physical morphology of the resulting sensor was characterized. Drawing inspiration from the BARC-III architecture [25], the device utilizes a serpentine magnetoresistive stripe configuration to optimize the effective sensing area (Figure 7a). This multi-layered architecture integrates the bottom GMR sensing layer with low-resistance Cr/Cu interconnects and Au bonding pads, while the trapezoidal MFCs are positioned directly above the sensing elements. For enhanced stability during liquid-phase bioassays, a dense SiO_2_ passivation layer encapsulates the structure, capped by a top Au film for biochemical functionalization.

Optical microscopy of the fabricated sensor (Figure 7b) reveals that the GMR stripes and MFC elements maintain precise spatial alignment. The sharp, well-defined boundaries of these microstructures suggest that the photolithography and lift-off sequences successfully translated the design specifications into the physical hardware. To quantitatively assess the sensor-to-sensor fabrication reproducibility, we randomly selected 20 sensing units from the finalized fabrication wafers. The measured zero-field base resistances tightly ranged from 9.45 to 9.79 kΩ. This highly uniform distribution corresponds to a relative standard deviation (RSD) of only 1.8%, demonstrating the high reliability and consistency of our microfabrication process.

**Figure 8:**
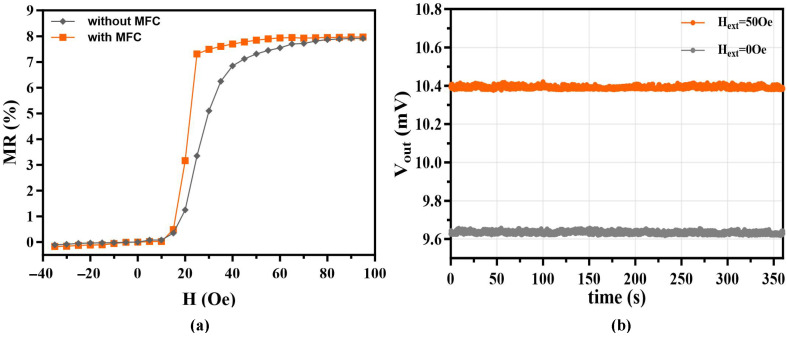
Magnetoresistive performance evaluation. (**a**) Comparison of MR transfer curves between the bare GMR sensor and the sensor with trapezoidal MFCs. (**b**) Real-time signal stability test of the sensor under 0 Oe and 50 Oe magnetic fields for 360 s.

We characterized the device’s magnetoresistive response utilizing a custom Helmholtz coil setup. As plotted in Figure 8a, integrating the trapezoidal MFCs noticeably steepens the transfer curve near the zero-field region compared to the bare sensor. The array delivers a peak magnetic sensitivity of 0.7024%/Oe within the linear range of 15 to 25 Oe.

In actual bioassays, the final readout relies on the interplay between the sensor’s intrinsic slope and the stray field emitted by the captured magnetic labels [20]. While the sensor itself is most responsive around 25 Oe, the physical volume of the 1-μm superparamagnetic beads is so small that they remain largely unsaturated at this weak bias. Consequently, the induced stray field they generate is too weak to produce a prominent signal shift. Increasing the external driving field to 50 Oe forces these magnetic labels into a significantly higher magnetization state. The resulting stronger bead-induced magnetic signal physically offsets the marginal drop in the sensor’s sensitivity slope, yielding an optimal practical signal-to-noise ratio (SNR) for the complete sandwich assay. Importantly, achieving this optimal bead magnetization on a bare sensor would require a high external field exceeding 70 Oe. Generating such intense fields in a point-of-care testing (POCT) setting demands bulky, power-hungry electromagnets, which severely compromises device portability and cost-effectiveness. The trapezoidal MFCs solve this by providing extreme localized magnetic gain, effectively amplifying the field at the sensor surface so that the beads can be sufficiently magnetized using a very small applied field of just 50 Oe.

To verify this 50 Oe operational setpoint, we recorded a 360 s continuous temporal scan (Figure 8b). The output maintained a highly stable baseline with negligible fluctuation under both 0 Oe and 50 Oe. This low-noise performance is primarily attributed to the device’s optimized base resistance (9.62 kΩ). Compared to earlier designs (e.g., 11.18 kΩ), this lower resistance inherently minimizes Joule heating ($P=I^{2}R$) under a constant excitation current, effectively suppressing thermal drift during continuous monitoring.

Table 3 explicitly benchmarks our trapezoidal architecture against conventional rectangular MFCs. Crucially, the 0.7024%/Oe magnitude perfectly preserves the absolute peak sensitivity observed in previous rectangular designs. However, rather than generating a severely localized magnetic peak, the optimized trapezoidal topology successfully extends this high-sensitivity response across a significantly broader spatial domain. It expands the field homogeneity ($H_{\mathrm{index}}$) to 81.8% and elevates the central magnetic gain to 169%. By eliminating the magnetic dead zones prevalent at the edges of rectangular concentrators, the structure maximizes the active biochemical sensing area. Every micrometer of the functionalized GMR surface is effectively utilized, guaranteeing that trace-level binding events are not lost to peripheral magnetic decay. Ultimately, this synergy of high spatial uniformity and a thermally quiet baseline equips the sensor for direct pathogen quantification in highly interfering matrices, such as mud, milk, and cider.

### 3.3. Feasibility and Stability Investigation

The biochemical functionalization and washing protocols inherently expose the sensor surface to various liquid reagents, including deionized water, alcohol, and PBS buffer. To rule out potential degradation of the passivation layer and the introduction of electrochemical background noise, we monitored the continuous baseline stability of the sensor across these environments prior to biological evaluations.

**Figure 9:**
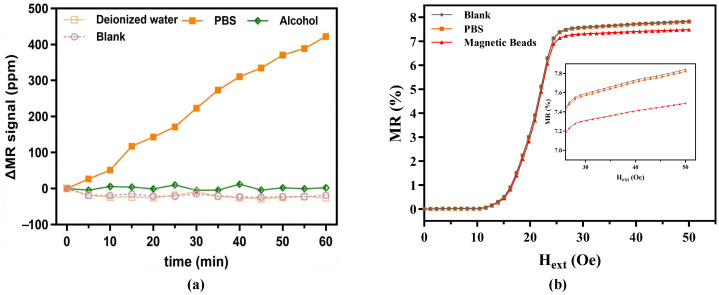
Stability testing and feasibility verification of the GMR biosensor. (**a**) Real-time baseline monitoring of the sensor in deionized water, alcohol, and PBS over 60 min. (**b**) MR transfer curves measured under blank, PBS, and magnetic bead conditions.

As illustrated in Figure 9a, continuous 1-h zero-field monitoring reveals the device’s robust tolerance to typical washing agents. Given the extremely minute variations, the MR signal shifts are expressed in ppm (1000 ppm = 0.1%). The dynamic responses in deionized water closely track the blank control, while the alcohol group exhibits negligible fluctuations (range <12 ppm), verifying that routine solvent washing preserves the structural integrity of the SiO_2_ passivation layer.

While the PBS buffer induced a slow monotonic drift accumulating to approximately 420 ppm over one hour—likely stemming from electric double-layer capacitance or micro-thermal effects associated with high ionic strength—this variation remains well below the typical 1000 ppm (0.1%) signal threshold generated by magnetic bead capture [30]. Considering that practical washing steps rarely exceed 10 min, this minor background drift is highly controllable and unlikely to distort the specific binding signals.

Beyond baseline stability, the sensor’s responsiveness to magnetic labels was evaluated by comparing the MR transfer curves under blank, PBS, and bead-dispensed conditions (Figure 9b). The overlapping curves of the blank and PBS groups further corroborate the SiO_2_ layer’s excellent shielding against electrochemical interference.

Conversely, the introduction of 1μm superparamagnetic beads triggers a distinct drop in the MR transfer curve (Figure 9b). This pronounced signal shift definitively validates the platform’s feasibility for tracking magnetic labels. Guided by the synergistic optimization of the sensor slope and bead magnetization established in Section 3.2, 50 Oe was consistently applied as the standard bias field for all subsequent quantitative bioassays.

### 3.4. Quantitative Detection and Real-Sample Analysis

**Figure 10:**
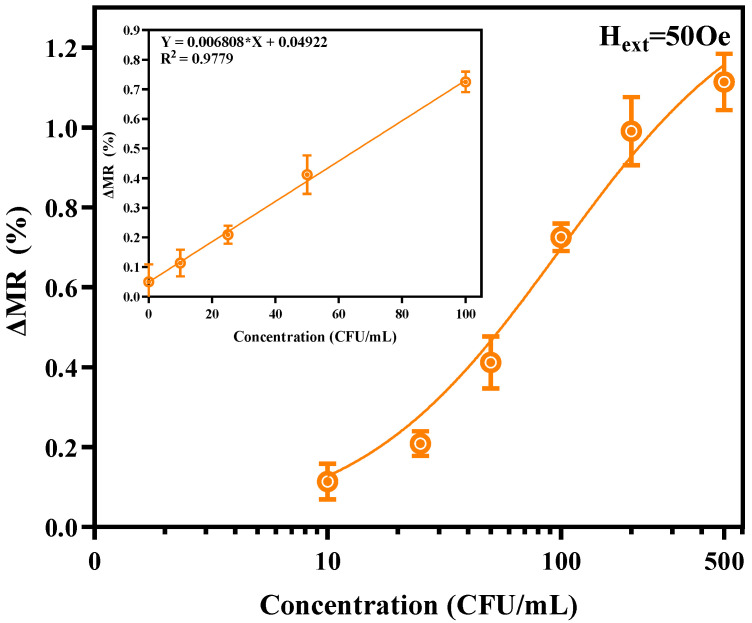
Quantitative detection of *B. anthracis*. The main plot displays the dose–response curve across a broad concentration range (0–500 CFU/mL) on a semi-logarithmic scale. The inset shows the precise linear fitting curve within the low-concentration dynamic range (0–100 CFU/mL). Error bars represent the standard deviation (SD) of three to five independent measurements (n=3–5).

As plotted in Figure 10, introducing inactivated *B. anthracis* at concentrations up to 500 CFU/mL triggered a steady, dose-dependent rise in the ΔMR readout on a semi-logarithmic scale. The low-concentration window (0–100 CFU/mL) displays excellent linearity (Y=0.00680X+0.04922, $R^{2}=0.9779$). Based on this calibration curve, the sensor establishes a practical limit of detection (LOD) of 10 CFU/mL.

It is crucial to note that while conventional rectangular MFCs can theoretically reach this 10 CFU/mL baseline under ideal buffer conditions, their inherent spatial field gradients frequently produce large measurement deviations. Because our trapezoidal geometry homogenizes the excitation field, it effectively eliminates the “magnetic dead zones” at the sensor periphery. This ensures that every captured bead contributes equally to the final readout, entirely independent of its random binding site across the active area. As a result, the proposed sensor accurately resolves trace targets down to 10 CFU/mL with reduced measurement deviation and excellent linearity. This performance effectively translates the LOD from a calculated theoretical boundary into a highly reliable quantitative standard.

**Figure 11:**
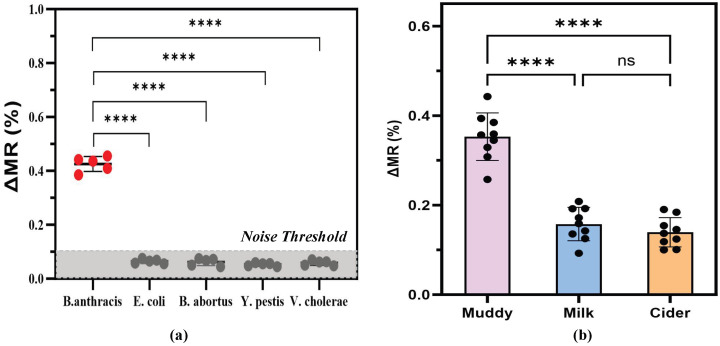
Specificity and complex matrix detection of *B. anthracis* (50 CFU/mL). (**a**) Specificity test against non-target bacterial strains. Statistical significance was evaluated using one-way ANOVA (**** p<0.0001 compared to the target group). (**b**) Detection results in complex matrices (muddy water, milk, and cider). While a significant signal attenuation was observed in milk and cider compared to muddy water (**** p<0.0001), no significant difference (ns) was found between the milk and cider groups. All error bars denote the standard deviation (SD) of independent measurements (n=5 for panel (**a**), and n=9 for panel (**b**)).

We assessed the sensor’s cross-reactivity by introducing 50 CFU/mL of non-target bacterial suspensions (*E. coli*, *B. abortus*, *Y. pestis*, and *V. cholerae*) to the functionalized interface. As Figure 11a illustrates, only the specific *B. anthracis* samples produced a distinct ΔMR drop. Statistical analysis via one-way analysis of variance (ANOVA) revealed a highly significant difference between the target *B. anthracis* group and all interfering species (**** p<0.0001, n=5). The resistance changes caused by the interfering species were negligible, essentially blending into the baseline noise. This clear separation in the readout confirms that the assembled double-antibody sandwich architecture maintains strict target recognition.

To determine how well the sensor handles real-world analytical environments, we directly inoculated 50 CFU/mL of *B. anthracis* into environmental mud, whole milk, and apple cider (Figure 11b). Extracting clean magnetic readouts from crude mud confirms that the dense SiO_2_ passivation effectively shields the underlying GMR stack from physical particulate accumulation and electrochemical screening. Crucially, the practical advantage of the trapezoidal sensing architecture becomes most evident in these highly heterogeneous mixtures. In complex fluids like mud or milk, particulate fouling often forces the captured magnetic beads into irregular, widely scattered spatial distributions. Conventional rectangular MFCs, hampered by peripheral magnetic dead zones, would fail to fully magnetize these displaced labels. In contrast, our extended uniform magnetic field consistently transduces the signal of the dispersed beads, successfully resisting the spatial blocking effects that typically paralyze conventional sensors. While the high viscosity and varying pH of the milk and cider caused a slight drop in the overall ΔMR amplitude compared to pure buffers (**** p<0.0001 versus muddy water), the specific pathogen signals remained unambiguously distinguishable from the background, showing remarkable stability with no significant variance between the milk and cider groups (ns, p>0.05, n=9). This robust anti-interference capability confirms that the optimized platform is highly suited for decentralized, on-site pathogen screening without requiring tedious sample purification.

Table 4 benchmarks the performance of the proposed trapezoidal MFC-GMR biosensor against conventional diagnostics and recent biosensing platforms for *B. anthracis*. Standard molecular techniques like PCR and real-time PCR typically reach a median limit of detection (LOD) of $4.3\times 10^{2}$ cells/mL. However, translating them to the field remains difficult due to long thermal cycling times (0.75–3.5 h) and the strict requirement for DNA extraction [31]. While lateral flow assays (LFAs) provide rapid visual results (<30 min) with minimal sample dilution, their LOD generally hovers around $10^{6}$ CFU/mL, which is insufficient for trace-level screening [32].

Optical methods using $Eu^{3+}/Tb^{3+}$ hybrids can achieve nanomolar-level detection of DPA biomarkers, but they require thermal lysis to release spore contents before the measurement [33]. Graphene FETs show an ultra-low LOD of 10 fg/mL for protective antigens in pure PBS buffers, yet these surface-charge-based sensors struggle with the Debye screening effect when exposed to highly ionic environmental samples [34]. More recent electrochemical immunosensors can detect whole spores in under 15 min without purification; even so, their LOD stalls at $10^{3}$ CFU/mL and testing has been limited to standard buffers [16].

Compared to these approaches, the trapezoidal MFC-GMR biosensor offers a more practical balance of sensitivity, speed, and minimal sample handling. Its LOD of 10 CFU/mL is roughly 43 times lower than the median PCR threshold, 100 times lower than recent electrochemical sensors, and five orders of magnitude better than LFAs. The entire assay takes about 40 min and avoids complex pretreatment steps like spore lysis, DNA extraction, or biomarker purification. Because the sensing mechanism is based on magnetic permeability rather than surface charge or optical transparency, it naturally bypasses Debye screening and optical scattering issues. This physical characteristic allows for the direct quantification of whole bacteria in complex, unpurified matrices like mud, milk, and juice, making it a straightforward and reliable tool for on-site biodefense monitoring.

## 4. Conclusions

In this study, we developed a GMR biosensor featuring trapezoidal magnetic flux concentrators (MFCs) for the rapid detection of *Bacillus anthracis*. Through systematic 3D computational modeling, we established the optimal MFC topology by defining its complete structural profile: a longitudinal length ($l_{c}$) of 1100 μm, a top width ($w_{\mathrm{tc}}$) of 750 μm, a bottom width ($w_{\mathrm{bc}}$) of 1000 μm, a film thickness ($t_{c}$) of 2 μm, and a critical sensing gap ($w_{g}$) of 450 μm. This precise geometric configuration mitigated peripheral flux saturation and achieved a practical balance between localized magnetic gain and spatial uniformity across the active sensing region. This homogenized magnetic field enabled the sensor to reliably detect bacterial concentrations down to an LOD of 10 CFU/mL.

Rather than merely pushing the limits of sensitivity in pure buffers, the primary contribution of this platform is its resilience in real-world analytical environments. Because the magnetic sensing mechanism is physically immune to the ionic Debye screening that restricts charge-based sensors, and unaffected by the optical scattering that limits colorimetric assays, the system can directly quantify whole cells in unpurified samples. Protected by a dense SiO_2_ passivation layer, the device maintained accurate readouts in highly heterogeneous matrices such as mud, milk, and apple cider, demonstrating robust tolerance to particulate fouling and pH variations.

By eliminating the need for complex sample pretreatments—such as thermal spore lysis, DNA extraction, or biomarker purification—the entire assay is completed in approximately 40 min. Operating efficiently under a low external driving field (50 Oe), this trapezoidal MFC-GMR biosensor provides a straightforward and highly pragmatic alternative to conventional laboratory-bound diagnostics. It presents a viable hardware solution for decentralized, on-site pathogen screening in biodefense and food safety applications.

## Figures and Tables

**Table 1:** List of key reagents and materials used in this study.

Category	Reagent/Material	Description/Cat. No.	Source/Supplier
Bacterial Strains	Inactivated *B. anthracis*	$10^{1}$–$10^{4}$ CFU/mL	Shanghai Yansheng Industrial Co., Ltd. (Shanghai, China)
	Inactivated *E. coli*	$10^{1}$–$10^{3}$ CFU/mL	Shanghai Yansheng Industrial Co., Ltd.
	Inactivated *B. abortus*	$10^{1}$–$10^{3}$ CFU/mL	Shanghai Yansheng Industrial Co., Ltd.
	Inactivated *Y. pestis*	$10^{1}$–$10^{3}$ CFU/mL	Shanghai Yansheng Industrial Co., Ltd.
	Inactivated *V. cholerae*	$10^{1}$–$10^{3}$ CFU/mL	Shanghai Yansheng Industrial Co., Ltd.
Antibodies	Anti-*B. anthracis* mAb	Capture probe (Cat. YS-MK1618)	Shanghai Yansheng Industrial Co., Ltd.
	Biotinylated anti-*B. anthracis* pAb	Detection probe (Cat. YS-DO118K)	Shanghai Yansheng Industrial Co., Ltd.
Magnetic Beads	Superparamagnetic Nanobeads	Dynabeads™ MyOne™ C1 (1.0 μm)	Thermo Fisher Scientific (Waltham, MA, USA)
Chemicals	N-Hydroxysuccinimide (NHS)	Thermo Scientific	Thermo Scientific (USA)
	PBS Buffer (pH = 7.4)	–	Shanghai Yuanye Biotechnology Co., Ltd. (Shanghai, China)
	11-Mercaptoundecanoic acid (11-MUA)	–	Shanghai Yuanye Biotechnology Co., Ltd.
	Bovine Serum Albumin (BSA)	–	Shanghai Yuanye Biotechnology Co., Ltd.
	1-Ethyl-3-(3-dimethylaminopropyl) carbodiimide (EDC)	–	Shanghai Yuanye Biotechnology Co., Ltd.
	Ethanol (Alcohol)	–	Sinopharm Chemical Reagent Co., Ltd. (Shanghai, China)
	Acetone	–	Sinopharm Chemical Reagent Co., Ltd.
Simulated Matrices	Apple Juice (Cider)/Whole Milk	NFC/Pasteurized	Local Supermarket

**Table 2:** Structural parameters and initial values for the design of the GMR model with trapezoidal magnetic flux concentrators.

Parameter	Description	Initial Value (μm)
$L_{m}$	Length of the magnetic sensitive region	460
$W_{m}$	Width of the magnetic sensitive region	430
$w_{g}$	Width of the air gap	450
$w_{\mathrm{tc}}$	Top width of the magnetic flux concentrator	460
$l_{c}$	Length of the magnetic flux concentrator	1200
$w_{\mathrm{bc}}$	Bottom width of the magnetic flux concentrator	1000
$t_{c}$	Thickness of the magnetic flux concentrator	2

**Table 3:** Quantitative comparison of magnetoresistive parameters and detection capabilities between different MFC geometries.

Parameter	Previous Work [23]	This Work
MFC Shape	Rectangular	Trapezoidal
Peak Sensitivity	0.71%/Oe	0.7024%/Oe
Magnetic Gain	125%	169%
Base Resistance	11.18 kΩ (Avg.)	9.62 kΩ (Avg.)
Field Homogeneity	Not systematically investigated	81.8% ($H_{\mathrm{index}}$)
LOD (Pure Buffer)	10 CFU/mL (*S. aureus*)	10 CFU/mL (*B. anthracis*)
Matrix Compatibility	PBS buffer	PBS buffer, mud, milk, and apple cider

**Table 4:** Performance comparison of various analytical techniques and biosensors for the detection of *Bacillus anthracis*.

Detection Method	Target Analyte	LOD	Assay Time	Sample Prep	Matrix Tested	Year	Ref.
PCR/Real-time PCR	DNA	$4.3\times 10^{2}$ cells/mL ^a^	0.75–3.5 h	DNA extraction	Soil, Water, Buffer	2009	[31]
Lateral Flow Assay (LFA)	Spores	$10^{6}$ CFU/mL	<30 min	Buffer dilution	Powders, Env.	2016	[32]
$Eu^{3+}/Tb^{3+}$ Hybrids	DPA (Biomarker)	1.06 nM	∼30 min	Thermal lysis (70 °C)	Aqueous buffer	2021	[33]
Graphene FET	Protective Antigen	10 fg/mL	∼20 min	Buffer exchange (Desalting)	PBS buffer	2023	[34]
Electrochemical sensor	Spores	$10^{3}$ CFU/mL	<15 min	Matrix removal	PBS buffer	2025	[16]
**Trapezoidal MFC-GMR**	**Whole Cells**	**10 CFU/mL**	**<40 min**	**None (Direct in matrix)**	**PBS, Mud, Milk, Cider**	**2026**	**This Work**

^a^ Median instrumental LOD derived from a meta-analysis of PCR methods.

## Data Availability

The original contributions presented in the study are included in the article; further inquiries can be directed to the corresponding author.
